# Experience of Spinal Surgery for the Over 70s During the COVID-19 Pandemic in a Tertiary Referral Centre in the United Kingdom and Ramifications for Future Management

**DOI:** 10.7759/cureus.51017

**Published:** 2023-12-24

**Authors:** Olivia Payton, Rohan Prakash, Adnan Shaikh, Md Faizul Hassan, Shahnawaz Haleem

**Affiliations:** 1 Spinal Surgery, Royal Orthopaedic Hospital, Birmingham, GBR

**Keywords:** risk, pathway, surgery, spine, covid-19

## Abstract

Introduction: The aim of this study was to assess the safety of our spinal surgery pathways for patients over the age of 70 years during the COVID-19 pandemic, to guide future management.

Methods: A retrospective, single-centre, observational cohort study of all patients over 70 years of age undergoing spinal surgery between June 1 to September 30, 2020, was performed. All patients were stratified by the British Orthopaedic Association (BOA) COVID-19 Patient Risk Assessment Tool.

Results: A total of 64 operations on 59 patients were performed. The BOA risk assessment placed 67.8% of patients (n=40) in the high or very high-risk category. A total of 60% of patients (n=36) were over 75 years old. All patients had at least one other comorbidity. Following our green, amber, and red pathways, we had no cases of post-operative COVID-19 on an average follow-up of 71 days.

Conclusion: Our study illustrates safe pathways for spinal surgery on patients over 70 years old during the first wave of COVID-19. Risk assessment tools should be used with caution, as age over 65 years was immediately medium-risk. This study would be a useful guide in the management of all elderly patients requiring surgery in the face of further COVID-19 variants or a similar pandemic.

## Introduction

Coronavirus disease (COVID-19), caused by severe acute respiratory syndrome coronavirus 2 (SARS-CoV-2), was first reported in the city of Wuhan in the Hubei province of China in December 2019 [1,2]. It was declared a pandemic by the WHO on March 11, 2020 [3]. Due to increased demand on the healthcare system in the United Kingdom (UK), in a letter to National Health Service (NHS) staff on March 17, 2020, hospitals in England were advised to stop all non-urgent elective care to provide additional capacity to the COVID-19 response [4,5]. This allowed for resources and staff to supplement the care of COVID-19 patients, whilst also preventing cross-infection between elective patients, staff, and COVID-19 patients [6]. Predictive modelling estimated that 28 million elective operations were cancelled or postponed worldwide due to the pandemic, during the peak 12 weeks of the first wave [7]. There was concern that operating on patients during the COVID-19 pandemic may carry a high mortality rate [8]. Age is a well-established risk factor for adverse effects from COVID-19 with over 90% of UK deaths in people over the age of 60 years [9]. Increasing age is associated with higher mortality with one study showing a 12-fold increased risk in the over 80 years age group compared to those aged 50-59 years [10]. This has also been supported by the COVIDSurg Collaborative’s work showing age over 70 years as an independent risk factor for mortality from peri-operative COVID-19, alongside male sex, major surgery, emergency surgery, and an American Society of Anaesthesiologists (ASA) grade of 3 or greater [11].

As hospitals were asked to restart elective work, there were concerns regarding the safety of performing surgery on elderly patients [12]. The Royal College of Surgeons (RCS) and the British Orthopaedic Association (BOA) prepared guidelines and prioritisation guidance to allow risk stratification and assist in operative decisions, supporting discussions on identified risk factors and the urgency of surgery [13,14]. Ultimately, however, this was a shared decision between patients and their surgeons made on an individual basis.

By 2021, there were approximately 10 million people in the UK awaiting urgent and elective surgery, with nearly 100,000 awaiting joint replacements [15]. Most orthopaedic elective patients were older with comorbidities and at risk of COVID-19 complications unless safe pathways were established [16]. Failure to institute such pathways and proceed with elective surgery would have increased the backlog and added to the risk profile and physical and mental distress of the patients. Our unit established COVID-19 pathways for elective spinal surgery at an early stage. This study was performed to assess the efficacy and safety of the spinal surgery services for patients over 70 years of age after the first UK lockdown in our specialist orthopaedic hospital. This will help guide future management as we remain susceptible to the emergence of new potentially highly virulent strains of COVID-19 going forward.

## Materials and methods

Our unit, Royal Orthopaedic Hospital NHS Foundation Trust, Birmingham, UK, is a tertiary referral centre for all orthopaedic services and a quaternary referral centre for bone tumours. It has no general medical or respiratory wards and no Intensive Treatment Unit (ITU). During the first lockdown, all elective, non-cancer surgery was stopped, with emergency and oncology work continuing. The study period reflects the hospital recommencing its elective surgery after the first wave. Once elective operating was restarted, there were three designated admission pathways. The green pathway was for all proven COVID-19-negative patients, with patients having two negative COVID-19 polymerase chain reaction (PCR) swabs and isolation for two weeks prior to admission. This pathway was primarily for elective cases. Filtering facepiece (FFP3) masks were only utilised for aerosol-generating procedures such as intubation. The amber pathway was used for suspected cases, where only one negative swab was taken, or the 14-day isolation period had not been completed. This pathway was utilised for emergency cases, including transfers from referring hospitals. All amber pathway cases were operated on at the end of the list or in a dedicated amber theatre. FFP3 masks were used during these cases. A red pathway was set up for COVID-19-positive patients, or those without any swabs. This pathway was only to be used in urgent cases where surgery could not be delayed. We had no spinal operations on this pathway during the study time frame, with any confirmed cases of COVID-19 being managed in a COVID-19 treatment hospital.

Patients were prioritised according to the RCS prioritisation guidelines [17]. All patients were stratified using the BOA risk assessment tool [14]. The risk of COVID-19 was explained to all patients pre-operatively and all patients appropriately consented to the additional risks of COVID-19 in the peri-operative period.

A retrospective, single-centre, observational cohort study for all patients over the age of 70 years undergoing spinal surgery between June 1, 2020, and September 30, 2020, was performed at our institution, a tertiary referral centre. The study was approved by the local audit committee prior to commencement. All elective and emergency cases were included, and case notes were reviewed. Electronic and paper records were reviewed. We checked for adherence to the green, amber, and red pathways set out by the trust. The BOA risk assessment tool was used to classify patients as low, medium, high or very high risk. We then reviewed the patients’ notes to assess whether there were symptoms of COVID-19, positive COVID-19 status, or complications post-operatively. Data was recorded using an electronic data collection spreadsheet. The COVID-19 status of patients was assessed pre-operatively with PCR ribonucleic acid (PCR-RNA) swabs. Patient comorbidities were recorded, along with length of stay.

## Results

We identified 65 operations on 60 patients over the age of 70 years undergoing spinal surgery between June 1 and September 30, 2020. There were 32 females and 28 males with an average age of 77.3 years (range 70-94 years) included in our study with 45 elective cases, 11 emergencies, five tumour cases, and four injection procedures (Table 1).

**Table 1:** Patient demographics

Characteristic	Patients
Male, n (%)	27 (45)
Mean BMI kg/m^2^	28 (20 – 41)
Mean age (years)	77.3
Age range (years)	n (%)
70-74	24 (40)
75-79	18 (30)
80-84	15 (25)
85-89	1 (1.7)
90-95	2 (3.3)
Comorbidities	n (%)
Hypertension	39 (66.1)
Diabetes mellitus	10 (16.9)
Cardiovascular disease	21 (35.6)
Renal disease	11 (18.6)
Pulmonary disease	19 (32.2)
History of cancer	7 (11.9)

All patients had at least one other comorbidity with hypertension being the most common (66.1%). The majority of patients had multiple comorbidities (n=57). The average length of hospital stay was 4.9 days (range 0-27). A total of 60% of patients were over the age of 75 years. We followed patients up for a mean of 71 days (range 25-173). The BOA risk assessment placed 67.8% of patients (n= 40) in the high or very high-risk category (Table 2).

**Table 2:** The American Society of Anesthesiologists (ASA) grading and the British Orthopaedic Association (BOA) risk stratification

ASA grade	n (%)
1	1 (1.5)
2	50(76.9)
3	14 (21.6)
4	0 (0)
BOA risk	n (%)
Low	0 (0)
Medium	19 (32.2)
High	29 (49.2)
Very high	11 (18.6)

All patients had evidence of at least one negative COVID-19 swab pre-operatively. Fourteen had only one negative swab on our electronic system and followed the amber pathway. Eight of these patients were out of the region and as per local policy had a PCR test at their local unit. The remaining six patients were emergency cases with surgery a clinical priority. A total of 46 patients followed the green pathway. No patients required the red pathway.

A total of 11 patients had a COVID-19 swab post-operatively, nine were routine as per hospital policy due to the patients being inpatients for seven days. Two were taken due to a neighbouring patient having a fever, as a precaution. All post-operative swabs were negative for COVID-19. We had no cases of post-operative COVID-19 during this timeframe, and no symptoms of COVID-19 were noted in routine follow-up.

Two patients sustained an incidental durotomy identified and managed intra-operatively with no complications. One patient had a hoarse voice following anterior cervical decompression and fusion (ACDF), which resolved. One patient had a cerebrovascular accident (CVA) two weeks post-operatively. One patient tested positive for carbapenemase-producing Enterobacteriaceae (CPE) post-operatively having been transferred from another hospital for emergency surgery without a CPE swab pre-operatively.

Seven patients required further surgery; four were operated on within the time frame of the study including evacuation of haematoma for two patients, re-operation due to infection in one patient, and re-operation due to metalwork failure in one patient. Three patients had further surgery outside the study period, one for infection and two had metalwork revised.

There was one mortality in an oncology patient (presumed spindle cell sarcoma) who developed bilateral pulmonary emboli after initial debulking surgery and passed away outside our study period after undergoing further surgery at another hospital.

## Discussion

It was predicted that between 885,286 and 1,028,733 patients were waiting for elective orthopaedic surgery in November 2020 [18], with a large proportion of these patients being elderly, and some patients reporting being in a “state worse than death” due to pain and decreased function [19]. The danger is that if risk stratification is over-cautious, patients may be inappropriately deemed too high-risk for surgery and may not be offered surgery [20]. It is imperative, therefore, that prioritisation is based on clinical need regardless of age.

Risk assessment tools should be used with caution to assess the danger of COVID-19 to patients. The BOA’s risk stratification placed all our patients as medium risk without any comorbidities, based purely on age greater than 65 years [14]. The addition of one comorbidity raises this to high risk. In the future, should we be faced with further strains of COVID-19, or indeed any future pandemic of a similar nature, we recommend such risk stratification tools are not used in isolation to decide whether to operate on patients, as they may unduly rule out patients based on age rather than genuine risk. This decision should be made on an individual basis as a shared decision with the patient. Daoust et al. warn against viewing age as having a linear effect on COVID-19-related outcomes [21].

Our study confirms that the utilisation of safe patient pathways as described can minimise the risk of COVID-19 in the peri-operative period. We believe that it was possible to provide safe spinal surgery to elderly patients during the COVID-19 pandemic provided strict pathways were adhered to, and a similar model can be followed in the future should the need arise. In our experience, this involved a two-week period of isolation prior to surgery, with two negative swabs taken 48-96 hours prior to planned admission. Once the patients were admitted to the hospital, they were managed on dedicated COVID-19-free wards. COVID-19 swabs were routinely repeated every seven days (if still admitted). Patients were advised to isolate for a further two weeks post-operatively.

With careful screening and segregation of patients, and operating at a stand-alone cold site, Lazizi et al. found that it was safe to resume elective operation in a time of endemic but low community prevalence [22].

We report no post-operative cases of COVID-19 despite some lengthy inpatient stays. We believe our pathways were effective in reducing this risk. It may also reflect the lower prevalence of COVID-19 in the older populations due to shielding and isolation at the time of the study. Complications were noted at a similar rate to pre-pandemic levels. Two patients (3.3%) had an incidental durotomy, which is comparable to the 3.8% noted in the large retrospective review prior to the pandemic by Guerin et al. [23]. 

To provide safe operative practices within the context of another COVID-19 pandemic, hospitals should develop patient scheduling and prioritisation systems [13]. These should be continuously updated to reflect the pandemic situation. Surgery should only take place after a full discussion of the risks and benefits of surgery including the risk of perioperative COVID-19 [15]. Within this discussion, the risks and benefits of postponing surgery should also be discussed.

Whilst our study demonstrates the safety of the pathways described, being a single-centre study based in the UK, the findings may not be generalisable to other healthcare systems. Furthermore, the results of our study must be viewed in context; going forward the levels of herd immunity in the population will fluctuate which will likely influence future pathways and policy. The sample only included patients undergoing treatment for spinal pathology and findings may not be generalisable to other surgical subspecialties. Our study highlights just one approach; the authors recognise that several other safe protocols may have been used at other institutions with success. 

## Conclusions

Our study confirms that appropriate pre-operative precautions in the form of self-isolation, negative swabs and the utilisation of individualised pathways lower the risks associated with surgical procedures and COVID-19 infection, despite the high-risk profile of the patients included. We believe that our approach utilising appropriate screening, isolation, and safe pathways should allow elective surgery for the elderly to proceed should we face further virulent strains of COVID-19 or indeed a similar pandemic. Our approach may also be of benefit to other surgical specialities.

## References

[1] Li Q, Guan X, Wu P (2020). Early transmission dynamics in Wuhan, China, of novel coronavirus-infected pneumonia. N Engl J Med.

[2] Zhu H, Wei L, Niu P (2020). The novel coronavirus outbreak in Wuhan, China. Glob Health Res Policy.

[3] (2023). Coronavirus disease 2019 (‎COVID-19)‎: situation report, 51. https://apps.who.int/iris/handle/10665/331475.

[4] (2023). Coronavirus » Important and urgent - next steps on NHS response to COVID-19. https://www.england.nhs.uk/coronavirus/documents/important-and-urgent-next-steps-on-nhs-response-to-covid-19/.

[5] Iacobucci G (2020). Covid-19: all non-urgent elective surgery is suspended for at least three months in England. BMJ.

[6] (2020). Global guidance for surgical care during the COVID-19 pandemic. Br J Surg.

[7] (2020). Elective surgery cancellations due to the COVID-19 pandemic: global predictive modelling to inform surgical recovery plans. Br J Surg.

[8] Lei S, Jiang F, Su W (2020). Clinical characteristics and outcomes of patients undergoing surgeries during the incubation period of COVID-19 infection. EClinicalMedicine.

[9] (2023). Statistics » COVID-19 daily announced deaths archive. https://www.england.nhs.uk/statistics/statistical-work-areas/covid-19-deaths/covid-19-daily-announced-deaths-archive/.

[10] Williamson EJ, Walker AJ, Bhaskaran K (2020). Factors associated with COVID-19-related death using OpenSAFELY. Nature.

[11] (2020). Mortality and pulmonary complications in patients undergoing surgery with perioperative SARS-CoV-2 infection: an international cohort study. Lancet.

[12] (2023). Coronavirus » Second phase of NHS response to COVID-19. https://www.england.nhs.uk/coronavirus/publication/second-phase-of-nhs-response-to-covid-19-letter-from-simon-stevens-and-amanda-pritchard/.

[13] (2023). Managing elective surgery during the surges and continuing pressures of COVID-19 — Royal College of Surgeons. https://www.rcseng.ac.uk/coronavirus/recovery-of-surgical-services/tool-7/.

[14] (2023). Re-starting non-urgent trauma and orthopaedic care: Full guidance. https://www.boa.ac.uk/resource/boa-guidance-for-restart---full-doc---final2-pdf.html.

[15] The Lancet Rheumatology (2021). Too long to wait: the impact of COVID-19 on elective surgery. Lancet Rheumatol.

[16] Price A, Shearman AD, Hamilton TW, Alvand A, Kendrick B (2020). 30-day outcome after orthopaedic surgery in patients assessed as negative for COVID-19 at the time of surgery during the peak of the pandemic. Bone Jt Open.

[17] (2023). Clinical guide to surgical prioritisation during the coronavirus pandemic — Royal College of Surgeons. https://www.rcseng.ac.uk/coronavirus/surgical-prioritisation-guidance/.

[18] Oussedik S, Zagra L, Shin GY, D'Apolito R, Haddad FS (2020). Reinstating elective orthopaedic surgery in the age of COVID-19. Bone Joint J.

[19] Scott CE, MacDonald DJ, Howie CR (2019). 'Worse than death' and waiting for a joint arthroplasty. Bone Joint J.

[20] Parvizi J, Gehrke T, Krueger CA, Chisari E, Citak M, Van Onsem S, Walter WL (2020). Resuming elective orthopaedic surgery during the COVID-19 pandemic: guidelines developed by the International Consensus Group (ICM). J Bone Joint Surg Am.

[21] Daoust JF (2020). Elderly people and responses to COVID-19 in 27 Countries. PLoS One.

[22] Lazizi M, Marusza CJ, Sexton SA, Middleton RG (2020). Orthopaedic surgery in a time of COVID-19: using a low prevalence COVID-19 trauma surgery model to guide a safe return to elective surgery. Bone Jt Open.

[23] Guerin P, El Fegoun AB, Obeid I (2012). Incidental durotomy during spine surgery: incidence, management and complications. A retrospective review. Injury.

